# A new species of *Paracreptotrema* (Digenea, Plagiorchiformes, Allocreadiidae) infecting two species of poeciliids in Río Malila of the Río Pánuco basin, Hidalgo, México, with a key to the species of the genus

**DOI:** 10.3897/zookeys.482.8144

**Published:** 2015-02-16

**Authors:** Christian E. Bautista-Hernández, Scott Monks, Griselda Pulido-Flores, Rafael Miranda

**Affiliations:** 1 Universidad Autónoma del Estado de Hidalgo, Centro de Investigaciones Biológicas, Apartado Postal 1-10, C.P. 42001, Pachuca, Hidalgo, México; 2 University of Navarra, Department of Zoology and Ecology, School of Sciencies, Irunlarrea n°1, E-31008 Pamplona, Navarra, Spain

**Keywords:** *Paracreptotrema*, *Xiphophorus malinche*, helminth parasite, endemic species, key

## Abstract

*Paracreptotrema rosenthali* **sp. n.** was discovered in the intestine of *Xiphophorus malinche* and *Pseudoxiphophorus jonesii*, collected from the headwaters of Río Malila, tributary of Río Conzintla, in the Río Pánuco basin, Hidalgo, México, during 2008–2009. The new species differs from the five known species of *Paracreptotrema* Choudhury, Pérez-Ponce de León, Brooks & Daverdin, 2006 by having vitelline follicles that extend from a level anterior to the pharynx to mid-testes, the seminal vesicle which is more extensively folded, and a wider cirrus sac. The new species resembles *Paracreptotrema heterandriae* in the length of its ceca, which surpasses the posterior margin of the ovary but do not reach the testes. A key to the species of *Paracreptotrema* is provided.

## Introduction

Despite an increase in our knowledge of the helminth parasites of the species of fish in México, Pérez-Ponce de León and Choudhury (2010) recently suggested that regions characterized by high biodiversity, such as the drainage basin of the Río Pánuco, need more intensive sampling. Their study indicated that the Poeciliidae, a family with many species endemic to México but with a limited range (Miller et al. 2005), could provide new information on the biodiversity of helminth parasites of freshwater fishes. *Xiphophorus malinche* Rauchenberger, Kallman & Morizot is such a poeciliid with a distribution restricted to the Río Pánuco basin. At present, it is known to inhabit only six isolated highland headwater streams (Culumber et al. 2011). Relatively little is known about the parasite communities of *Xiphophorus malinche*; however, a recent study compared parasite communities between two populations of this species and reported differences in the helminth communities that the authors attributed to geographic isolation (Bautista-Hernández et al. 2014b). As part of that study, an undescribed species of *Paracreptotrema* Choudhury, Pérez-Ponce de León, Brooks & Daverdin, 2006 was recovered in one of these populations; it is described herein and a key to the known species is presented.

## Materials and methods

Adult specimens of *Xiphophorus malinche* (60 individuals; May 2008 to July 2009) and *Pseudoxiphophorus jonesii* (Günther, 1874) (*sensu* Agorreta et al. 2013) (= *Heterandria jonesii*) (30 individuals; August 2012) were collected from the Río Malila, a tributary of the Río Conzintla, northeastern Hidalgo, México. Fish were collected using minnow traps, brought live to the laboratory of the Centro de Investigaciones Científicas de las Huastecas Aguazarca (CICHAZ) field station in Calnali, Hidalgo, and examined within 24 h after capture. Fish were fixed in ethyl alcohol (EtOH 96%) for confirmation of their identification. Trematodes were collected live, killed in warm water and fixed for 24 h in alcohol-formalin-acetic acid. Specimens were stained with Mayer’s carmalum or Delafield’s hematoxylin, mounted whole in Canada balsam, and examined using bright-field and differential interference contrast optics. Illustrations were made with a drawing tube attached to the microscope; measurements are given in micrometers (µm) and are expressed as the range of measurements followed by the mean ± standard deviation in parentheses. Comparisons of other members of the genus with the new species are made from the original descriptions, but full data on each species from all published works are given in Table 1; reported measurements are given exactly as in the original work because all of the original specimens were not available to be re-measured.

**Table 1.** Comparison of morphological characteristics of the five species described as *Paracreptotrema*. Data for *Paracreptotrema blancoi* (México), *Paracreptotrema blancoi* (Costa Rica), *Paracreptotrema mendezi* and *Paracreptotrema heterandriae* taken from Choudhury et al. (2006) and Salgado-Maldonado et al. (2012). Note: measurements are given exactly as in the original work with the same precision as reported and presented as the range followed by the mean.

	*Paracreptotrema blancoi*	*Paracreptotrema blancoi*	*Paracreptotrema mendezi*	*Paracreptotrema profundulusi* (text)**
**Body length**	465–732 (519)	500–850 (688)	680	600–990 (788)
**Maximum width**	200–387 (263)	250–450 (349)	310	287–500 (364)
**Oral sucker length × width**	70–90 (82) × 82.5–110 (90.4)	100–155 (126.7) × 100–155 (126.2)	100 × 120	102–150 (125) × 112–177 (137)
**Ventral sucker length × width**	162.5–207 (175.9) × 125–210 (173.8)	120–175 (153.2) × 130–205 (174)	170 × 170	165–250 (201) × 145–225 (189)
**Sucker ratios**				
**Length**	1.7–2.4 (1:2.1)	1.2–1.21 (1:1.2)	1:1.4’	1.5–2.0 (1:1.6)
**Width**	1.4–2.5 (1:1.9)	1.2–1.7 (1: 1.4)		1.1–1.5 (1:1.4)
**Pharynx length × width**	25–50 (40) × 30–62.5 (47.2)	40–60 (50) × 45–75 (57)	50 × 60	37–62 (48) × 37–70 (49)
**Ovary length × width**	37.5–77.5 (51.5) × 25–75 (40.7)	55–110 (86.2) × 35–62.5 (69.2)	27 × 74	47–125 (72) × 50–125 (90)
**Left testis length × width**	45–75 (64.2) × 37.5–70 (52.5)	90–170 (132) × 70–120 (94.7)	180 × 90	87–175 (124) × 75–125 (93)
**Right testis length × width**	42.5–87.5 (63.7) × 35–62.5 (52.5)	89–167 (134) × 74–115 (95.1)	170 × 90	87–175 (122) × 60–112 (92)
**Cirrus sac length × width**	62.5–137.5 (83.2) × 30–50 (37.5)	– × 35–62.5 (52)	100 × 60	92–175 (135) × 37–95 (67)
**Eggs length × width**	45–60 (52) × 25–37.5 (32.5)	52.5–62.5 (55.4) × 32.5–42.5 (38.5)	46 × 37	50–62 (57) × 25–37 (31)
**Locality**	Río Papagayo basin, Guerrero, México	Área de conservación, Guanacaste, Costa Rica	Lake Gatun, Panama	Río Tehuantepec basin, Oaxaca, México
**Host**	*Profundulus punctatus* (Profundulidae)	*Priapichthys annectens* (Poeciliidae)	*Brachyrhaphis episcopi* (Poeciliidae)	*Profundulus punctatus* (Profundulidae)
**Reference**	Salgado-Maldonado et al. 2011	Choudhury et al. 2006	Sogandares-Bernal 1955	Salgado-Maldonado et al. 2011

	*Paracreptotrema profundulusi* (table)*	*Paracreptotrema heterandriae*	*Paracreptotrema rosenthali*
**Body length**	675–990 (820)	625–1,050 (783)	720–940 (830)
**Maximum width**	287–500 (380)	175–375 (252)	350–550 (417)
**Oral sucker length × width**	115–137 (124) × 117–150 (133)	100–160 (121) × 87–150 (113)	105–160 (125) × 130–175 (140)
**Ventral sucker length × width**	175–250 (205) × 145–225 (186)	112–195 (155) × 117–217 (163)	170–203 (203) × 180–225 (205)
**Sucker ratios**			
**Length**	1.5–2.0 (1:1.7)	0.9–1.6 (1:1.3)	1:1.6
**Width**	1.1–1.5 (1:1.3)	1.2–1.7 (1:1.4)	1:1.5
**Pharynx length × width**	37–62 (48) × 37–70 (51)	45–67 (54) × 50–75 (59)	45–55 (52) × 38–70 (56)
**Ovary length × width**	47–125 (82) × 75–125 (98)	55–112 (76) × 42–112 (72)	63–135 (104) × 98–145 (111)
**Left testis length × width**	87–155 (122.4) × 62–112 (92)	87–150 (119) × 52–125 (75)	105–188 (136) × 68–168 (96)
**Right testis length × width**	87–162 (123.4) × 60–112 (99)	87–150 (116) × 57–112 (79)	110–188 (137) × 50–155 (88)
**Cirrus sac length × width**	100–175 (141) × 37–87 (69)	100–145 (114) × 20–35 (28)	75–110 (91) × 68–103 (80)
**Eggs length × width**	52–60 (57) × 25–30 (27.8)	70–75 (72.5) × 35–41 (40)	47–70 (52) × 25–45 (32)
**Locality**	Río Tehuantepec basin, Oaxaca, México	Río la Antigua upper basin, Xalapa, Veracruz, México	Río Conzintla, Malila, Hidalgo
**Host**	*Profundulus punctatus* (Profundulidae)	*Heterandria bimaculata* (Poeciliidae)	*Xiphophorus malinche* (Poeciliidae)
**Reference**	Salgado-Maldonado et al. 2011	Salgado-Maldonado et al. 2012	Present study

* Data taken from the table displayed in Salgado-Maldonado et al. (2011).

** Data taken from the original description in text (Salgado-Maldonado et al. 2011).

## Results

### Systematic account Family Allocreadiidae (Looss, 1902)

#### Genus Paracreptotrema

Taxon classificationAnimaliaPlagiorchiidaAllocreadiidae

Choudhury, Pérez-Ponce de León, Brooks & Daverdin, 2006

##### Type species.

*Paracreptotrema blancoi* Choudhury, Pérez-Ponce de León, Brooks & Daverdin, 2006

#### Paracreptotrema rosenthali sp. n.

Taxon classificationAnimaliaPlagiorchiidaAllocreadiidae

http://zoobank.org/CD6087D6-0AA7-40B1-B06B-0756B52E2681

Figure 1

##### Type material.

Holotype (CNHE 9263), 3 paratypes (CNHE 9264 to 9266), and 3 paratypes (HWML 75051 to 75054).

##### Other material examined.

*Paracreptotrema blancoi* Choudhury, Pérez-Ponce de León, Brooks & Daverdin, 2006 (CNHE–5315, Costa Rica; CNHE–7682, México); *Paracreptotrema heterandriae* Salgado-Maldonado, Caspeta-Mandujano & Martínez-Ramírez, 2012 (CNHE–8242); *Paracreptotrema mendezi* (Sogandares-Bernal, 1955) Choudhury, Pérez-Ponce de León, Brooks & Daverdin, 2006 (HWML–22193, 22194); and *Paracreptotrema profundulusi* Salgado-Maldonado, Caspeta-Mandujano & Martínez-Ramírez, 2011 (CNHE–7684).

##### Type host.

*Xiphophorus malinche* Rauchenberger, Kallman & Morizot (Poeciliidae). Vouchers deposited in MZNA fish collection, University of Navarra, Spain (Galicia et al. 2014).

##### Type locality.

Río Malila, tributary of Río Conzintla, northeastern Hidalgo, México (20°44'N; 98°43'W).

##### Site in host.

Intestine.

##### Other host.

*Pseudoxiphophorus jonesii* (Günther, 1874) (*sensu* Agorreta et al. 2013; = *Heterandria jonesii*). Vouchers deposited in MZNA fish collection, University of Navarra, Spain (Galicia et al. 2014).

##### Prevalence.

In *Xiphophorus malinche*, 7 of 88 infected (7.9%). In *Pseudoxiphophorus jonesii*, 1 of 36 infected (2.77%).

##### Etymology.

The species is named in honor of Gil G. Rosenthal, Department of Biology, Texas A&M University, College Station, Texas, and co-founder of the CICHAZ field station, for his friendship, contributions to the knowledge of species of *Xiphophorus*, and in recognition of his efforts to promote science in the Huasteca region of México.

##### Description.

**Figure 1.**
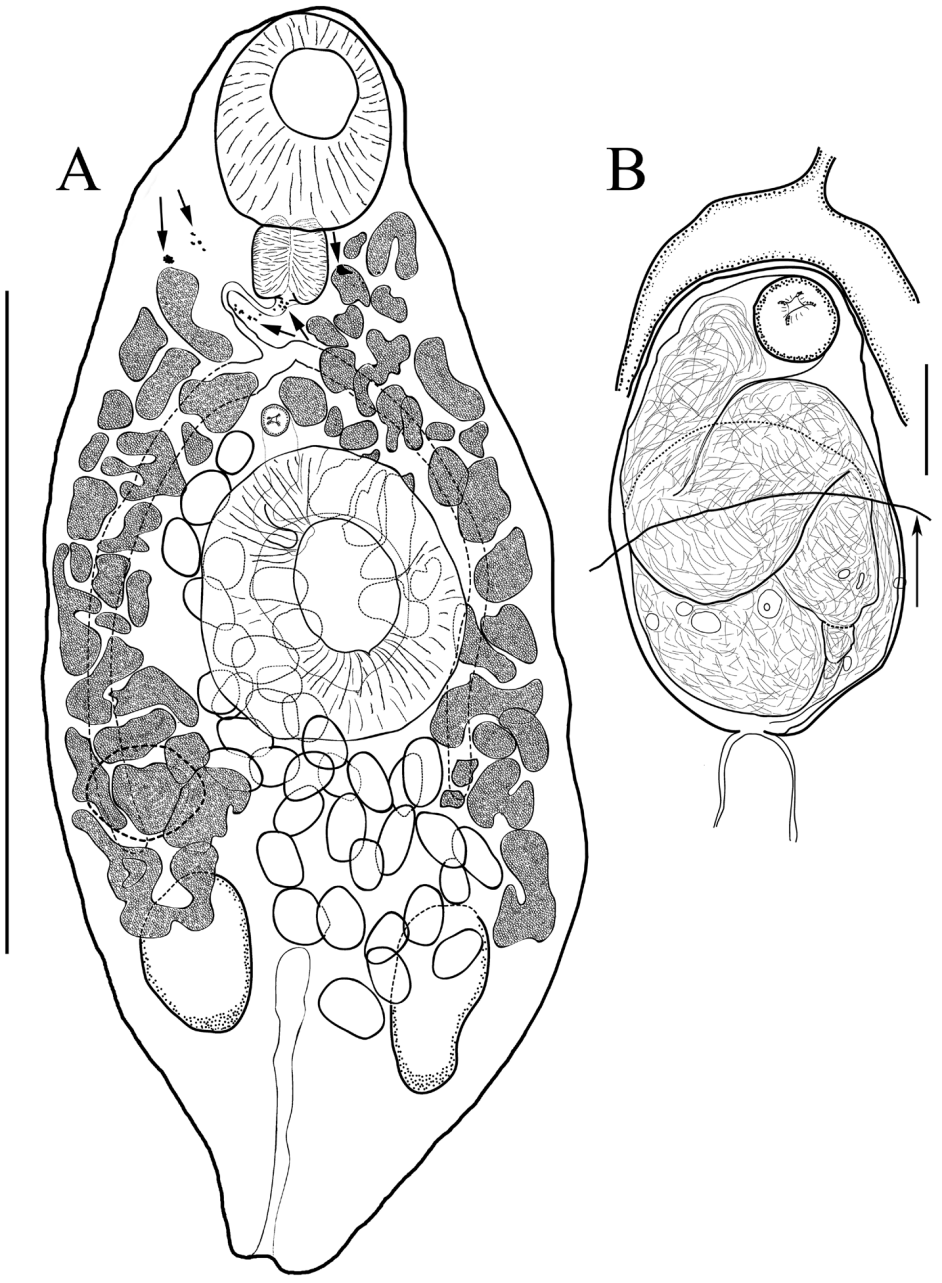
*Paracreptotrema rosenthali* sp. n. **A** Ventral view of holotype; arrows indicate fragments of eyespot pigment **B** Cirrus sac; arrow indicates anteriormost margin of acetabulum. Scale bars: **A** = 250 µm; **B** = 25 µm.

[Based on 8 specimens] Body 720–940 (830 ± 83 n = 7) long, robust, aspinose. Anterior end rounded; body 350–550 (417 ± 66 n = 7), widening gradually, reaching maximum width at level of middle to posterior margin of acetabulum, terminating in narrower posterior end. Few, small, pigment spots in forebody. Oral sucker wider than long, subterminal, 105–160 (125 ± 20 n = 7) long, 130–175 (140 ± 16 n = 7) wide, with 2 papillae on posterior margin and several papillae along outer edge; opening subterminal, antero-ventrally directed. Average length of acetabulum, 170–230 (203 ± 18 n = 8) slightly greater than width, 180–225 (205 ± 15 n = 7), strongly muscular, sunken, visible externally by its rounded opening; tegument of acetabulum with fine striations radiating outward from acetabular opening. Ratio of length of oral sucker to length of acetabulum 1:1.4–1:2.0 (1:1.6) and width of oral sucker to width of acetabulum 1:1.3–1:1.7 (1:1.5). Prepharynx absent. Pharynx muscular, well developed, 45–55 (52 ± 4, n = 6) long, 38–70 (56 ± 11 n = 6) wide. Esophagus short, winding, 45–75 (57 ± 16, n = 3) long, 5–10 (n = 2) wide. Cecal bifurcation short distance anterior to acetabular margin. Ceca, mostly obscured by vitelline follicles, extend posterior to acetabulum, 725 (right side) to 800 (left side) long (n = 1), following curve of body, just surpassing posterior margin of ovary but not reaching testes (Fig. 1). Ovary 63–135 (104 ± 25 n = 8) long, 98–145 (111 ± 16 n = 7) wide, entire, dextral (4 of 8) or sinistral (4 of 8), 350–600 (435 ± 85 n = 8), 48%–64% (54% ± 6% n = 7) from anterior end of body, overlapping posterior margin of acetabulum in some specimens. Mehlis’ gland comprised of loose aggregate of gland cells, 25–63 (43 ± 12 n = 7) long, 25–68 (48 ± 15 n = 7) wide, partially obscured by vitelline follicles but more visible from dorsal perspective. Seminal receptacle postovarian, muscular. Laurer’s canal not observed. Uterus with loop extending to posterior margin of testes. Vitellarium follicular, in 2 lateral fields, each consisting of a roughly-formed double row of follicles, 1 row more lateral and 1 partially overlapping acetabulum dorsally. Vitelline follicles extend from level anterior to pharynx to middle of testes, to posterior margin of testes in some specimens. Lateral fields of vitellarium loosely confluent antero-dorsal to acetabulum, dorsally overlapping ceca, lateral margins of acetabulum, ovary, and testes. Follicles consist of granular vitelline cells; vitelline ducts connect to large median vitelline reservoir filled with vitellocalcyl cells. Eggs number about 20 to 40, well developed, ovoid, operculate, 40–70 (52 ± 7 n = 40) long, 25–45 (32 ± 4 n = 40) wide. Testes 2, irregular in form but not lobed, longer than wide, post-equatorial, posterior to and separated from ovary. Right testis 110–188 (137 ± 30 n = 8) long, 50–155 (88 ± 37 n = 7) wide, anterior margin 460–700 (571 ± 83 n = 8), 63%–78% (71% ± 5% n = 7) from anterior end of body. Left testis 105–188 (136 ± 32 n = 8) long, 68–168 (96 ± 38 n = 7) wide, anterior margin 490–760 (591 ± 88 n = 8), 69%–84% (73% ± 6% n = 7) from anterior end of body. Cirrus sac elongate, median, dorsal, 75–110 (91 ± 18 n = 3) long, 68–103 (80 ± 20 n = 3) wide, containing coiled tubular seminal vesicle that occupies most of space in cirrus sac (Fig. 1b). Genital pore median, between cecal bifurcation and anterior margin of acetabulum. Excretory vesicle I-shaped, tubular, narrow, reaching anteriorly to or slightly beyond level of mid-testes. Excretory pore terminal.

### Remarks

The genus *Paracreptotrema* includes four species: *Paracreptotrema blancoi* Choudhury, Pérez-Ponce de León, Brooks & Daverdin, 2006, *Paracreptotrema mendezi* (Sogandares-Bernal, 1955), *Paracreptotrema profundulusi* Salgado-Maldonado, Caspeta-Mandujano & Martínez-Ramírez, 2011, and *Paracreptotrema heterandriae* Salgado-Maldonado, Caspeta-Mandujano & Vázquez, 2012. The specimens of *Paracreptotrema rosenthali* sp. n. from *Xiphophorus malinche* share the features established in the original concept of the genus (Choudhury et al. 2006). In general, there are five primary features that can be used to distinguish *Paracreptotrema rosenthali* sp. n. from the extant species: the shorter length of the ceca, the extent of the vitelline follicles, the extensive folding of the seminal vesicle, the width of the cirrus sac, and the extension (area occupied) of the uterus. *Paracreptotrema rosenthali* resembles *Paracreptotrema mendezi*, *Paracreptotrema blancoi*, and *Paracreptotrema profundulusi* in having a well-developed cirrus sac, but the new species stands out by having a seminal vesicle that is more extensively folded and the cirrus sac which is wider than those of the other three taxa. *Paracreptotrema rosenthali* sp. n. and *Paracreptotrema heterandriae* have ceca that extend past the ovary but not to the testes; however, in the latter species the body is longer and narrower than that of *Paracreptotrema rosenthali* sp. n. The vitellarium of the new species extends from a level anterior to the pharynx to the middle of the testes, and in some specimens reach but do not pass the posterior margin of the testes, and the follicular rows partially overlap the acetabulum dorsally. In *Paracreptotrema mendezi*, the vitellarum extends posteriorly from the oral sucker but does not pass the anterior margin of the testes. In *Paracreptotrema blancoi* it extends from the cecal bifurcation to the anterior edge of the testes. In *Paracreptotrema profundulusi*, the vitellarium extends to the postesticular area, and in *Paracreptotrema heterandriae* the vitelline follicles extend from the cecal bifurcation to the posterior margin of the testes. The extent of the uterus of *Paracreptotrema rosenthali* sp. n. is similar to the uterine distribution of *Paracreptotrema blancoi* and *Paracreptotrema heterandriae* in that the uterus extends to the posterior margin of the testes, often filling the post-testicular area; in *Paracreptotrema profundulusi* the uterus is mostly pretesticular. The mean size of the eggs of *Paracreptotrema rosenthali* sp. n. (52 long by 32 wide) is similar to that of *Paracreptotrema blancoi* (55 by 39) and *Paracreptotrema profundulusi* (57 × 31); the mean egg size of *Paracreptotrema mendezi* (46 × 37) is smaller and that of *Paracreptotrema heterandriae* (72 × 40) is larger. The number of eggs in the uterus ranged from 20–40 among the specimens of the new species; the specimens of *Paracreptotrema blancoi* that we examined had fewer than 10 eggs and those of the three other species that we observed had from 8–24 eggs in the uterus.

## Discussion

Geographic barriers play an important role in the isolation of fish populations and their helminth fauna (Pérez-Ponce de León and Choudhury 2010; Salgado-Maldonado et al. 2011; Bautista-Hernández et al. 2014b). Four of the five species of *Paracreptotrema*, including the one described in this paper, are parasites of poeciliid fish. Choudhury et al. (2006) suggested that *Paracreptotrema* spp. might be parasites exclusive to poeciliids in the Neotropical region. However, Salgado-Maldonado et al. (2011) described *Paracreptotrema profundulusi* from and reported *Paracreptotrema blancoi* in species of the Profundulidae, arguing that this was evidence that *Paracreptotrema* spp. could have a closer relationship with freshwater members of the Profundulidae than with the Poeciliidae because of the restricted distribution of the latter family in Central America. The geographic distribution of *Profundulus* is restricted to hydrological basins of Central America, extending northward only to the Isthmus of Tehuantepec (southeastern México), so the co-occurrence of the two species of *Paracreptotrema* in those fish could be due to recent contact between different host populations. The finding of the new species does not offer insights into the co-speciation of the members of the genus; i.e. the origin of each species and whether they originated in poeciliids or profundulids. For this reason, a phylogeny of the group is needed, ideally combined with a hypothesis regarding the taxa that host these species. Additionally, in cases where the localities of each species of helminth are widely separated, further studies are needed to verify the limits of the distribution of each.

The distribution of *Xiphophorus malinche* is restricted to the more northern Hidalgo anticline, separated from southern populations of fish by the barrier range of the Mexican plateau (Kallman and Kazianis 2006), so it is not clear how the population of *Paracreptotrema rosenthali* sp. n. is linked to those species of Central America. Consistent with hypotheses regarding the orogeny and isolation of headwater populations, Bautista-Hernández et al. (2014b) reported differences in parasite communities between two populations of *Xiphophorus malinche* (Chicayotla and Malila) that are separated only by two mountain ridges. Specifically, the Malila population was infected with three species of helminth, whereas the Chicayotla population was infected with four species. Our finding a new species restricted to the Malila population further supports the importance of host biogeographic factors with regard to the structure of helminths communities. Although helminth diversity is affected by the restricted distribution of their host, further studies are needed to evaluate the familial host specificity of species of *Paracreptotrema*. *Paracreptotrema mendezi* was collected from fish living in a lake but all other known species are from stream- and river-dwelling populations of fish; whether or not this factor is important for our understanding of the ecological relationships of the members of the genus is still unknown.

The papillae on the oral sucker were difficult to discern on our specimens. Two papillae on the posterior margin of the sucker were visible on some specimens, but only some of the papillae along outer edge were visible on a few specimens; thus, no papillae were included in the figure. We could discern several papillae along the outer edge of the oral sucker in specimens of *Paracreptotrema blancoi*, *Paracreptotrema profundulusi*, and *Paracreptotrema heterandriae*, but the entire complement of papillae was not visible in any specimens we examined. Study of specimens using scanning electron microscopy will be necessary for a full assessment of the number of papillae present, but the number of specimens available at this time is not sufficient for such a study.

All known species of *Paracreptotrema* have an oral sucker that is wider than long (Table 1). The new species is not different in this respect. However, one specimen we collected, the holotype (unfortunately), had an oral sucker longer than wide (Fig. 1). This specimen was processed differently to any of the others, and it was one of six specimens from single-worm infections, but it is the only one with the different sucker size ratio. Even with that worm removed from the comparison, the oral sucker of *Paracreptotrema rosenthali* sp. n. is the largest of the known species. Similarly, the average length of the acetabulum was greater than the width, but in some worms this was reversed.

The presence of Laurer’s canal has been reported for the four previously known species. We were not able to discern the canal in specimens of the new species. The limited material precluded mounting of specimens in a more favorable position for observations of this structure, and no specimens were available for histological study. The populations of fish from which the specimens were collected are limited in size and fragile, and this helminth has not been found in other populations of fish close to the locality (Bautista-Hernández et al. 2014a; Bautista-Hernández et al. 2014b), but the presence of Laurer’s canal needs to be confirmed by future studies.

Razo-Mendivil et al. (2014) provided molecular evidence that *Paracreptotrema heterandriae* is a member of the Allocreadiidae, affording strong support for the familial relationship previously suggested by Choudhury et al. (2006) and Salgado-Maldonado et al. (2012). A more inclusive molecular study of the new species would provide additional information on the relationships of this species with *Paracreptotrema heterandriae* and the other members of the genus. Molecular evidence would also provide confirmation of the specific identification of the putative species which have been identified to date. Morphological characters, some of which can vary intraspecifically, have been the primary features used to identify species; molecular techniques could verify or falsify the appropriateness of the morphological features that have been used.

### Key to the identification of species of *Paracreptotrema*

**Table:** 

1	Ceca do not surpass the anterior margin of the testes	**2**
–	Ceca surpass the anterior margin of the testes	**4**
2	Anterior margin of the vitelline follicles does not reach the anterior margin of the pharynx	***Paracreptotrema heterandriae***
–	Anterior margin of the vitelline follicles surpasses the anterior margin of the pharynx	**3**
3	Uterus extends to the posterior margin of the hindbody	***Paracreptotrema mendezi***
–	Uterus does not extend to the posterior margin of the hindbody	***Paracreptotrema rosenthali***
4	Vitellarium extends posterior to the testes	***Paracreptotrema profundulusi***
–	Vitellarium does not extend into the region posterior to the testes	***Paracreptotrema blancoi***

## Supplementary Material

XML Treatment for Paracreptotrema

XML Treatment for Paracreptotrema rosenthali
